# Interval‐induced metabolic perturbation determines tissue fluid shifts into skeletal muscle

**DOI:** 10.14814/phy2.14841

**Published:** 2021-04-27

**Authors:** Mirko Mandić, Mikael F. Forsgren, Thobias Romu, Per Widholm, Patrik Sundblad, Thomas Gustafsson, Eric Rullman

**Affiliations:** ^1^ Department of Laboratory Medicine Division of Clinical Physiology Karolinska Institutet, and Unit of Clinical Physiology Karolinska University Hospital Stockholm Sweden; ^2^ AMRA Medical AB Linköping Sweden; ^3^ Department of Health, Medicine and Caring Sciences Linköping University Linköping Sweden; ^4^ Center for Medical Image Science and Visualization (CMIV) Linköping University Linköping Sweden; ^5^ Department of Radiology, and Department of Health, Medicine and Caring Sciences Linköping University Linköping Sweden

**Keywords:** ^31^P‐MRS, edema, HIIT, MRI, plasma volume, SIT

## Abstract

Intense interval exercise has proven to be as effective as traditional endurance exercise in improving maximal oxygen uptake. Shared by these two exercise regimes is an acute reduction in plasma volume, which is a suggested stimulus behind exercise‐induced increases in blood volume and maximal oxygen uptake. This study aimed to link exercise‐induced metabolic perturbation with volume shifts into skeletal muscle tissue. Ten healthy subjects (mean age 33 ± 8 years, 5 males and 5 females) performed three 30 s all‐out sprints on a cycle ergometer. Upon cessation of exercise magnetic resonance imaging, ^31^Phosphorus magnetic resonance spectroscopy and blood samples were used to measure changes in muscle volume, intramuscular energy metabolites and plasma volume. Compared to pre‐exercise, muscle volume increased from 1147.1 ± 35.6 ml to 1283.3 ± 11.0 ml 8 min post‐exercise. At 30 min post‐exercise, muscle volume was still higher than pre‐exercise (1147.1 ± 35.6 vs. 1222.2 ± 6.8 ml). Plasma volume decreased by 16 ± 3% immediately post‐exercise and recovered back to – 5 ± 6% after 30 min. Principal component analysis of exercise performance, muscle and plasma volume changes as well as changes in intramuscular energy metabolites showed generally strong correlations between metabolic and physiological variables. The strongest predictor for the volume shifts of muscle and plasma was the magnitude of glucose‐6‐phosphate accumulation post‐exercise. Interval training leads to large metabolic and hemodynamic perturbations with accumulation of glucose‐6‐phosphate as a possible key event in the fluid flux between the vascular compartment and muscle tissue.

## Graphical abstract

Here we show that skeletal muscle metabolic perturbation after a bout of high intensity interval exercise correalltes with plasma‐volume drop and a corresponding increase in muscle volume.

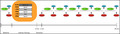

## 1 INTRODUCTION

There has been a steep rise in the interest regarding interval exercise regimes in recent years. Much of the attention stems from the intriguing effectiveness of interval training in improving maximal aerobic capacity ($\dot{V}O_{2}\text{max}$) and associated cardiovascular and peripheral physiological factors. The two most common forms of interval exercise are high intensity interval training (HIIT) and sprint interval training (SIT). As interval training can be modified in numerous ways by manipulating variables such as intensity, duration, and rest to work ratio, the terminology in the literature is rather inconsistent (Weston et al., 2014). In short, HIIT is interval training performed at an intensity eliciting 80–95% of maximal heart rate, whereas SIT is performed at an intensity corresponding to $\dot{V}O_{2}\text{max}$ and above, including all‐out efforts (MacInnis & Gibala, 2017).

When comparing interval training with more traditional endurance exercise (TEE) performed continuously and at lower intensities, it is evident that the nature of the two training modes is vastly different. Still, a large number of studies demonstrate that interval training induces similar metabolic adaptations as TEE (Burgomaster et al., ,,^2005^, ^2008^; Gist et al., 2014; Milanović et al., 2015; Rakobowchuk et al., 2008). At the molecular level, both HIIT and SIT stimulate skeletal muscle signaling pathways, suggested to play a key role in peripheral adaptations, to a comparable degree as TEE (Egan et al., 2010; Gibala et al., 2009; Little et al., 2011; Norrbom et al., 2004). Moreover, comparable changes in $\dot{V}O_{2}\text{max}$ have generally been noted (Gist et al., 2014; Milanović et al., 2015; Phillips et al., 2017), which is intriguing since aerobic capacity is mainly dependent on central hemodynamic factors.

A tight relationship exists between total blood volume, cardiac output and $\dot{V}O_{2}\text{max}$ (Celsing et al., 1987; Convertino, 2007; Convertino et al., 1980; Kjellberg et al., 1949). Plasma volume expansion accounts for nearly all the increase in blood volume during the first 2–3 weeks after onset of exercise (Fellmann, 1992; Gore et al., 1992). This is followed by a later increase in erythrocyte volume until reaching equilibrium, i.e., restored hematocrit value corresponding to pre‐training levels (Sawka et al., 2000). Albumin is accountable for ~75% of the oncotic pressure in plasma and is therefore a crucial factor in the maintenance of plasma volume (Gillen et al., 1991). It has been shown that increased plasma albumin accounts for ~85% of the total plasma protein content during exercise‐induced hypervolemia (Convertino et al., 1980). Thus, an increase in blood volume must be preceded by an increased synthesis of albumin (Tsutsumi et al., 1993). It has been hypothesized that reduced plasma volume acts to stimulate mechanisms leading to increased albumin synthesis and total plasma volume (Convertino et al., 1980, 1983; Röcker et al., 1976; Rothschild et al., 1972; Yang et al., 1998). Given that a reduction in plasma volume is seen with both HIIT, SIT (Lundvall et al., 1972; Metcalfe et al., 2015) and TEE (Convertino et al., 1983), it is possible that this comparable degree of plasma flux explains why increases in $\dot{V}O_{2}\text{max}$ are possible with both interval training and TEE training.

Breakdown of muscle glycogen leads to a hypertonic intramyocellular environment through accumulation of metabolites such as hydrogen ions (H^+^), inorganic phosphate (P_i_), lactate, and glucose‐6‐phosphate (G6P). Interval training causes a 20–30% reduction in muscle glycogen content (Esbjörnsson‐Liljedahl et al., 1999; Gibala et al., 2009; Parolin et al., 1999), and the muscle glycogen breakdown occurs predominantly during the first 15 s of a sprint (Metcalfe et al., 2015; Parolin et al., 1999). Accumulation of these metabolites has been demonstrated to lead to an influx of water in skeletal muscle (Raja et al., 2006; Ward et al., 1996; Watson et al., 1993), which provides a possible mechanism for plasma volume loss noted with interval exercise. Tissue water content can be assessed using magnetic resonance imaging (MRI) by measuring transverse relaxation time (T2), where increases in accumulated metabolites and tissue water content results in an increased signal intensity on T2‐weighted MR images (Fisher et al., 1990; Fleckenstein et al., 1988; Price & Gore, 1998; Price et al., 1995). By combining MRI measurements with ^31^P magnetic resonance spectroscopy (^31^P‐MRS) it is possible to measure pH, inorganic phosphate (P_i_), G6P and other intramuscular energy derivatives.

The aim with this study was to characterize sprint‐induced plasma flux in relation to muscle metabolic perturbation where we hypothesize that 1) 3 × 30 s all‐out sprints cause a robust increase in muscle volume and reduction in plasma volume, and that 2) the changes in metabolic perturbation are correlated with the changes in muscle volume.

## 2 METHOD

### 2.1 Exercise protocol

**FIGURE 1:**
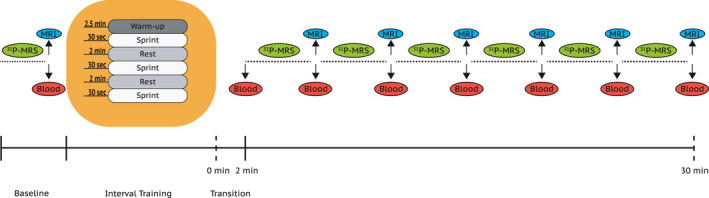
Study overview. PCr, pH, ATP and G‐6‐P concentrations were sampled continuously over the 30 min period post‐exercise (^31^P‐MRS, dotted line). Only interrupted by the collection of T2 and Dixon sequences every 5 min (MRI). Blood samples were taken in connection to the MRI‐scans. During recovery from exercise the ^31^P‐MRS acquisition was interjected for acquisition of standard MRI sequences at 8, 14 and 20 min and finally at 23, 27, and 30 min post‐exercise

The exercise protocol was comprised of three 30 s all‐out sprints against a breaking force equivalent to 7.5% of the subject's body weight. Each sprint was separated by 2 min of low intensity cycling. Subjects were given strong verbal encouragement during each interval. The sprints were performed on a mechanically braked cycle ergometer (Ergomedic 894E). The cycle setup was placed just outside the MRI‐suite and subjects were moved from the cycle ergometer to the MRI and then positioned in a predefined supine position before the post‐measurements started. This procedure was practiced with each subject prior to the experiment to minimize loss of time between exercise completion and the first measurement. The surface coil used for ^31^P‐MRS was positioned at the vastus lateralis and the area of the coil was marked to ensure identical placement during both baseline and post‐measurements. The transfer of the subjects from the cycle ergometer to the MRI took approximately 120 s, counting the time from exercise cessation until start of data acquisition. Phosphocreatine (PCr), pH, adenosine triphosphate (ATP), and G6P concentrations were acquired at baseline, and continuously from completion of exercise until 30 min post‐exercise with interruptions of approximately 45 s every 5 min for collection of Dixon‐sequences for assessment of muscle volumes. Exact time points can be found in Figure 1. Blood was sampled before, immediately after exercise and thereafter in 5 min intervals up to 30 min post‐exercise. Plasma volume drop was calculated using the Dill and Costill equation (Dill & Costill, 1974).

### 2.2 Dixon imaging

Using the built‐in transmit/receive body coil a standard T1‐weighted gradient echo sequence with a 2‐point Dixon reconstruction (Dixon VIBE) was acquired in the axial plane centred around the phosphorus surface coil. Acquisition parameters were TE1 1.15 ms, TE2 2.3 ms, TR 3.8 ms, FOV 50 × 35 cm, and voxel size 1.2 × 1.2 × 4 mm with a scan time of about 1 min. Fat, water, in‐phase and out‐of‐phase images were reconstructed on the scanner console. The images were then imported into AMRA Researcher where semi‐automated volume analysis were performed as described earlier (Mandić et al., 2020). The pre‐exercise baseline reading was acquired immediately after a 5‐min ^31^P‐MRS acquisition. All image acquisitions were performed on a Philips Ingenia 3T.

### 2.3 ^31^P‐MRS acquisition

**FIGURE 2:**
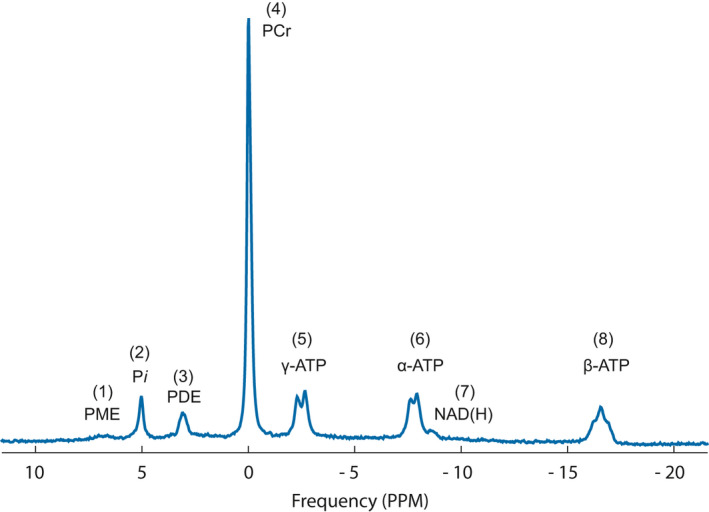
The spectra show the baseline ^31^P‐MRS for a representative subject in the cohort. The resonances in the spectrum corresponds to; (1) Phosphomonoesters, also glucose‐6‐phosphate in the recovery phase, (2) Inorganic phosphate, (3) Glucose phosphates and Phosphodiesters, (4) Phosphocreatine, (5,6,8) α‐,β‐, and γ‐ nucleotide triphosphate (mainly ATP), (7) NAD(H)

Two different spectroscopic acquisition protocols were used; one for baseline acquisition, with high signal‐to‐noise ratio (SNR), and one for rapid dynamic measurements during the recovery phase following the exercise. For both protocols, a 31P transmit‐receive surface loop coil provided by the scanner manufacturer (‘P‐140’; Philips) with a diameter of 14 cm was used for spectroscopic measurements. The coil was manually tuned and matched before the 31P MRS acquisitions. A 42° block pulse was used for the spin excitation. The baseline measurements were performed using 15 s repetition time (TR), 153.4 μs echo time (TE), 3 kHz bandwidth, 2048 data points, 16 averages, 2 dummy scans, and a synthesizer frequency of 51.717 MHz. To increase the temporal resolution the recovery phase spectra were acquired with a 2 s TR and no spectral averaging, a total of 150 single spectra were acquired with no dummy scans. For post‐processing of the ^31^P‐MRS data, jMRUI (Naressi et al., 2001) was used with the AMARES algorithm (Vanhamme et al., 1997) for relative quantification of the resonances. Prior to quantifying the dynamic data, the first two FIDs of each sequence were dropped (i.e., dummy scans) thereafter the data was averaged in blocks of ten, reducing the temporal resolution to 20 s to enhance SNR. Phosphocreatine (PCr) was used as a chemical‐shift reference with assignments obtained from literature as previously described (Gerdle et al., 2013, 2020) and showed for a baseline dataset in Figure 2. In short, phosphomonoesters (PME) were assigned to phosphoethanolamine and phosphocholine, in the dynamic phase this resonance also includes glucose‐6‐phosphate (G6P; details below; Haan et al., 2003); inorganic phosphate (P_i_) and PCr were defined as singlets. The phosphodiester (PDE) resonance was assigned to glycerophosphoethanolamine and glycerophosphocholine. In addition, the resonance corresponding to NAD(H) was also observed. Finally, the nucleotide triphosphate (NTP‐Mg, mainly adenosine triphosphate [ATP]) resonances were assigned and interpreted as α‐,β‐, and γ‐NTP as previously reported (Gerdle et al., 2013, 2020). pH was estimated in the spectra using the modified Henderson–Hasselbach equation to assess the chemical shift difference between P_i_ and PCr (with pK_A_ = 6.77, δ_HA_ = 3.23 ppm, and δ_A_ = 5.70 ppm) as described previously (De Graaf, 2007) using the built in functionality in jMRUI.

The baseline acquisition served to establish the steady‐state resting concentrations of the metabolites of interest where absolute concentrations of metabolites were estimated using ATP as reference, assuming an intramuscular concentration of 8.2 mM or 5.5 mmol/kg wet weight muscle (Harris et al., 1974). During the recovery phase after exercise, it was assumed that all metabolites, including ATP/NTP, were perturbed and therefore the rate of recovery over time for each metabolite was calculated, rather than their respective ratio to ATP. G6P was estimated by calculating the differences between the quantified PME resonances and the last acquisition (Equation 1). While others have calculated the difference by subtracting baseline spectra from the dynamic sets (Haan et al., 2003), this was not feasible in the current setting since (1) the exercise bouts could not be performed within the MR bore and (2) the chemical shift differences due to the very high post‐exercise intra‐myocellular lactate concentrations.

(1) $$\Delta \text{G6P}\left(t\right)=\frac{\text{PME}(t)}{\text{PME}(t=\text{end})}.$$

The dynamics of PCr and P_i_ were estimated by relating each timepoint to the last (Equation 1; assuming that the last time point was acquired in pseudo‐steady state). The increase of G6P was also assessed in relation to NTP (‘G6P:NTP’). Correction for relaxation differences of the resonances due to the not fully relaxed spectra in the dynamic acquisition was performed by correcting each quantised resonance with Equation 2.

(2) $$\text{Correction}\;\text{factor}=\frac{1}{1‐\exp\left(\frac{‐\text{TR}}{T_{1}}\right)}\frac{1}{\exp\left(\frac{‐\text{TE}}{T_{2}}\right)},$$

TR and TE are specific to the ^31^P‐MRS acquisition protocol and *T*_1_ and *T*_2_ are relaxation times of the resonances being corrected. The relaxation times were taken from the literature (Bogner et al., 2009) assuming β‐NTP and PME to have similar *T*_2_ characteristics as α‐NTP and PDE, respectively.

### 2.4 Statistics

All averages are given as geometrical mean and standard deviation if not stated otherwise; standard deviation is corrected for within‐subjects effect (Morey, 2008). Principal Component Analysis was carried out with variables scaled to unit variance using FactoMineR version 2.0 on the statistical platform R version 3.5.3. Where appropriate, normality was tested using the Kolmogorov‐Smirnov test, and pairwise t‐tests were used to contrast pre‐ and post‐exercise measures. One‐way repeated measures ANOVA was used with Tukey HSD as post‐hoc tests. The parameters describing rate of recovery post‐exercise for the different ^31^P‐MRS spectra (i.e., metabolites) were calculated by exponential curve‐fitting on an individual basis and the time‐constant, rate of recovery at *t*_0_ and concentration at *t*_0_ (as fraction of the final concentration) was retained. Subject‐by‐subject curve‐fittings can be found in Figure S1.

### 2.5 Ethical approval

All subjects were given oral and written information about the study before giving informed consent to participate. The study was approved by the Swedish Ethical Review Authority.

## 3 RESULTS

### 3.1 Baseline characteristics

Ten healthy subjects participated in the study, the group consisted of 5 females and 5 males (age 33 ± 8 years, height 175.2 ± 7.4 cm, weight 74.1 ± 11.6 kg). At baseline, all subjects had normal hemoglobin and hematocrit values, 137.5 ± 6.5 g/L and 41 ± 2% respectively. The participants were moderately active but were not involved in any structured exercise programs.

### 3.2 Exercise and workload

**FIGURE 3:**
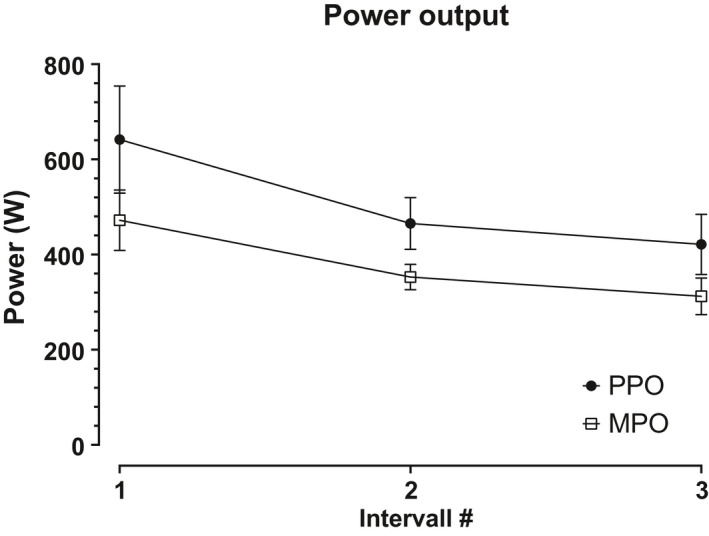
Peak power output and mean power output for the three intervals done before post‐exercise data acquisition. Both peak‐ and mean power output decreased significantly from interval 1 to interval 2. No significant change was observed from interval 2 to interval 3. Values expressed as mean ± SD

For each individual and each exercise bout, power output was recorded and summarized into peak power output (PPO) and mean power output (MPO). Peak and mean power for bout 1 was 642 ± 113 and 472 ± 64 W which decreased to 465 ± 54 and 353 ± 27 W in bout number 2. (*p *< 0.010). The peak and mean power output did not change significantly between bouts 2 and 3, 421 ± 63 and 312 ± 38 W, respectively (Figure 3).

### 3.3 Plasma volume/Hemoconcentration

**FIGURE 4:**
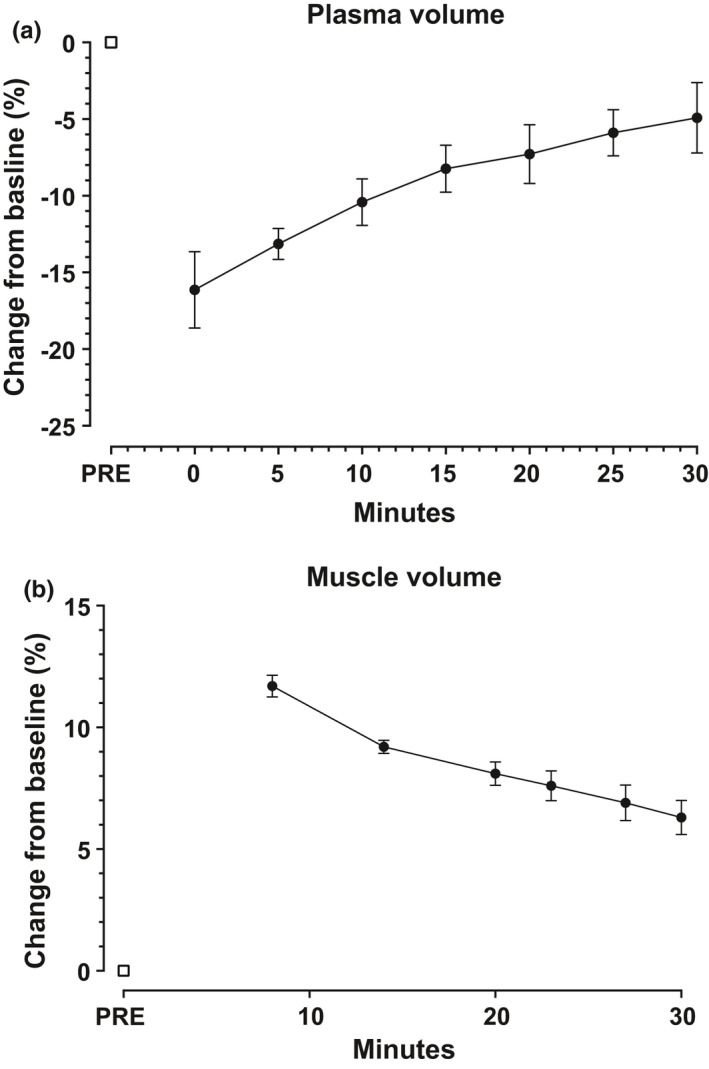
(a) Changes in plasma volume expressed as change in percent compared to baseline. Measurements were taken at baseline (PRE) and directly after exercise (0) and at 5, 10, 15, 20, 25, and 30 min post‐exercise. (b) Changes in muscle volume expressed as change in percent compared to baseline. Post‐exercise measurements were taken at 8, 14, 20, 23, 27, and 30 min. Values expressed as mean ± SD

Hemoglobin concentration increased from 137.5 ± 6.5 g/L at baseline to 155.3 ± 9.2 g/L immediately after exercise (*p *< 0.001). Thereafter it decreased by an average of 0.5 g/min, reaching 140.9 ± 10.9 g/L 30 min post‐exercise. Plasma volume dropped by 16 ± 3% immediately post‐exercise and recovered back to −5 ± 2% at 30 min post‐exercise (Figure 4a).

### 3.4 Muscle volume

At baseline, muscle volume of the quadriceps was 1147.1 ± 35.6 ml, which increased by an average of 12% to 1283.3 ± 11.0 ml 8 min post‐exercise. At 14 min post‐exercise, the quadriceps volume had decreased to 1254.7 ± 5.8 ml; thereafter it followed a linear downward slope over the subsequent four measurements. At 30 min post‐exercise, the muscle volume had decreased to 1222.2 ± 6.8 ml, but it was still significantly higher than pre‐exercise (Figure 4b). Similarly, the muscle volume of the hamstring musculature increased from 1085.3 ± 29.3 ml to 1175.9 ± 7.5 ml 8 min post‐exercise. Likewise, the hamstring volume decreased to 1147.3 ± 6.7 ml 30 min post‐exercise, which also remained higher compared to the pre‐exercise volume.

### 3.5 ^31^P‐MRS estimation of metabolites

**FIGURE 5:**
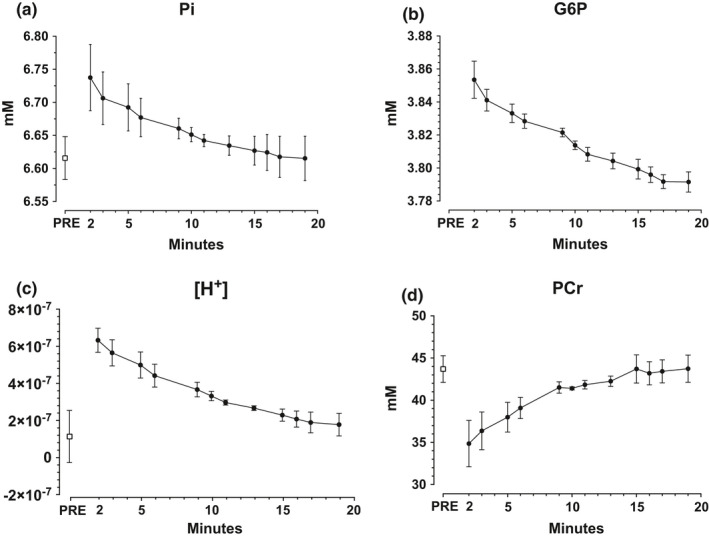
Panel showing intra‐muscular metabolites measured with ^31^P‐MRS at baseline (PRE), 2, 3, 5, 6, 9, 10, 11, 13, 15, 16, 17, and 19 min post‐exercise. (a) Free phosphate (Pi), (b) Glucose‐6‐phosphate (G6P) note that baseline measurement for G6P is not available due to low signal at baseline, (c) [H^+^] concentration, (d) Phosphocreatine (PCr). Values expressed as mean ± SD

Free phosphate (Pi) concentration was 6.6 ± 0.03 mM at pre‐exercise and 6.7 ± 0.05 mM at 2 min post‐exercise. The concentration of Pi decayed with a time‐constant of 362 ± 198 s and with a rate of decay of 0.03 ± 0.08%/s (Figure 5a). Due to the low concentration, we were not able to obtain data on the baseline concentration of G6P. 2 min post‐exercise G6P concentration was 3.85 ± 0.01 mM and recovered thereafter with a time‐constant of 415 ± 70 s corresponding to a rate of 0.004 ± 0.002%/min (Figure 5b). At baseline, muscle pH was 6.94 ± 0.21. It dropped to 6.22 ± 0.06 at 2 min post‐exercise and recovered to 6.79 ± 0.05 at 19 min post‐exercise, with a recovery time constant of 960 ± 509 s corresponding to 0.10 units/min. Corresponding changes as H^+^ is shown in Figure 5c. The baseline pre‐exercise phosphocreatine concentration was 43.7 ± 1.6 mM. At 2 min post‐exercise PCr was 34.9 ± 2.7 and it thereafter recovered with a time‐constant of 372 ± 126 s, which corresponded to a recovery rate of 5.4 ± 0.8%/min (Figure 5d).

### 3.6 Workload, muscle volume and metabolite relationships

**FIGURE 6:**
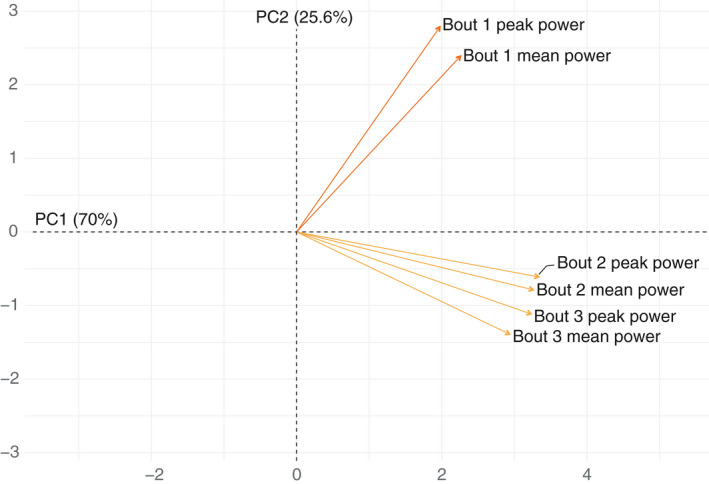
Principal component analysis (PCA) for measured power outcomes. The PCA shows a very high correlation between the variables were PC1 and PC2 explains approximately 95% of the total variance. The variables cluster into two distinct and uncorrelated categories, power output for bouts 2 and 3 along PC1, and power output for bout 1 along PC2

**FIGURE 7:**
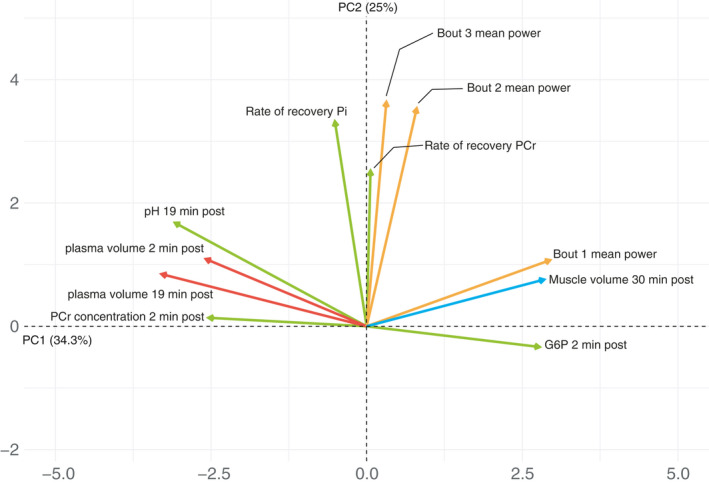
Principal component analysis (PCA) including both intra‐muscular metabolites and power output outcomes where all variables with significant correlations are showed. PCr depletion, pH drop, and G6P accumulation are together with changes in muscle and plasma volume mutually correlated and cluster along PC1. PCr recovery and power output variables from bouts 2 and 3 are also mutually correlated but cluster along PC2 indicating no significant correlations with the other variables

**FIGURE 8:**
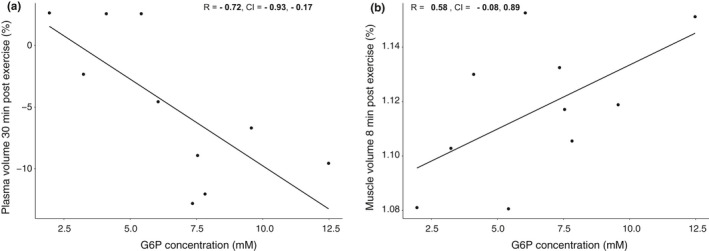
To confirm the relationship between muscle and plasma fluid shift and G6P accumulation post‐exercise as indicated by the principal component analysis a linear regression model was fitted: There was a negative correlation between G6P and plasma volume drop (*R* = −0.72) and a corresponding positive correlation between G6P accumulation and muscle volume increase following exercise (*R* = 0.58). Changes in PME‐intensity corresponding to an accumulation of G6P. Plasma and muscle volumes are relative delta changes compared to pre‐exercise

The association between the different intensity measures over the three exercise bouts was explored using PCA, which revealed that all intensity measures were highly correlated within each bout of exercise (i.e., a high peak power was associated with a higher mean power output). All intensity measures from bout numbers 2 and 3 were in turn highly correlated but the intensity measures of bout number 1 were largely independent from the other two. Thus, the power output of each individual can be largely described in terms of two components, PC1, mainly described by the power output from bout numbers 2 and 3, and PC2 described by the power output of bout number 1 (Figure 6). Subsequently a new principal component analysis was performed analyzing exercise performance, muscle and plasma volume, as well as ^31^P‐MRS‐data. This revealed a high degree of mutual correlation between the investigated variables and a substantial portion of the variance could be summarized into the first (34.3%) and second component (25%; Figure 7). Variables with a significant correlation to the first two components are shown in Figure 7, loadings of all variables are shown in Table S1. PCr depletion, pH drop, and G6P‐accumulation together with changes in muscle volume and the decline in plasma volume were all significantly correlated with PC1 whereas PCr‐recovery and the power output variables measured in exercise bouts 2 and 3 were significantly correlated with PC2. The metabolite most significantly correlated with PC1 was changes in PME corresponding to an increase in G6P, together with muscle volume and plasma volume. This was further supported when the mutual correlation of these factors was investigated (Figure 8).

## 4 DISCUSSION

This study used a novel approach to estimate the exercise‐induced fluid shift into skeletal muscle tissue and to relate this muscle volume change to the metabolic perturbation induced by exercise. Following three bouts of intense interval exercise, a drop in plasma volume and an increase in thigh muscle volume was observed. Coupled with these changes, measurements using ^31^P‐MRS reveled an increase in Pi and G6P together with a simultaneous decrease in pH and PCr. Accumulation of G6P and the increase in muscle volume was highly correlated. Exercise power output during the second and third exercise bout was consistent whereas the first bout was largely independent from the other two. Principal component analysis revealed that changes in muscle and plasma volume co‐varied with PCr depletion, pH drop and G6P‐accumulation whereas power‐output during the second and third exercise bout co‐varied with post‐exercise PCr‐recovery.

Previously it has been suggested that the increase in tissue fluid with exercise is due to an accumulation of fluid in the intra‐cellular compartments (Raja et al., 2006). This has been explained by contraction‐induced increases in intra‐muscular osmolality and capillary filtration pressure (Lundvall et al., 1972; Stick et al., 1990). Using the Du Bois formula to calculate body surface area and the normative tables of plasma volume from Hurley, we can estimate a baseline plasma volume of 3027 ± 428 ml in the current study (Du Bois & Du Bois, 1989; Hurley, 1975). The drop in plasma volume in the present study amounted to 16 ± 3%, which corresponded to ~484 ml of plasma loss. This change is of similar magnitude as earlier reports exploring exercise of a longer duration (Greenleaf et al., 1979; Lundvall et al., 1972; Sjøgaard & Saltin, 1982). Furthermore, the loss matches the increase in muscle tissue water we observed in the presumably most active muscles when performing interval exercise on a bike (Silva et al., 2016). The plasma volume recovery was faster than what was observed for the muscle tissue. While plasma volume was close to be completely restored 30 min post‐exercise muscle volume was still substantially higher compared to baseline measurements. This discrepancy has been reported earlier and has been suggested to be due to extravascular fluid exchange between inactive tissues and contracting skeletal muscle tissue (Lundvall et al., 1972; Stick et al., 1990). Plasma fluid loss into the contracting muscle increases the plasma osmolality, which is one of the driving forces behind fluid outflow from inactive tissue. Based on the vasoconstriction in inactive tissue during exercise, a reduced filtration pressure has also been suggested to contribute (Lundvall et al., 1972; Stick et al., 1990). Thus, it can be assumed that plasma volume can be replenished from sources other than the active muscle post‐exercise and thereby attenuate the drop in plasma volume, thus protecting venous return and cardiac stroke volume (Wingo et al., 2012). Regardless of these effects, our data clearly show a substantial plasma volume drop and corresponding muscle fluid infiltration after intense interval exercise.

During more conventional type of endurance exercise, the exercise‐induced loss of plasma volume leads, in an intensity‐dependent fashion, to hypertonic hypovolemia, which is highly correlated to increased releases of aldosterone and renin‐angiotensin (Convertino et al., 1981). These hormones are assumed to drive the development of hypotonic hypervolemia occurring post‐exercise (Convertino et al., 1980; Green et al., 1984). Importantly, oncotic pressure in plasma is the major regulator of albumin synthesis (Rothschild et al., 1972), which has also been reported to increase following short term high intensity exercise (Yang et al., 1998). Altogether, the current data support our hypothesis that three very brief bouts of high intensity interval training induce similar reduction in plasma volume as has been previously reported with more conventional types of endurance training. We therefore suggest that increased blood volume and $\dot{V}O_{2}\text{max}$ in response to traditional endurance training as well as interval training plausibly occurs through the same mechanism.

As stated above, exercise‐induced increases in muscle fluid and volume are assumed to be at least partly due to osmosis, mainly because of the increased intramuscular osmolality driven by exercise‐induced intramuscular metabolic perturbations (Lundvall et al., 1972; Stick et al., 1990). Thus, metabolic perturbation is assumed to be one of the key processes behind exercise‐induced fluid inflow to the exercising skeletal muscle. In the present study, the G6P concentration measured post‐exercise explained muscle volume changes to the greatest extent: A positive correlation was observed between changes in PME‐intensity corresponding to an accumulation of G6P and muscle volume expansion and a negative correlation was seen between G6P and plasma volume drop. This is an indication that during sprint exercise the accumulation of lactate and shift in NADH/NAD+ratio leads to a decrease in the flux‐rate of metabolite though the glycolysis leading to an accumulation of G6P due to a mismatch in the flux though glycolysis and the rapid breakdown of glycogen. Based on the mechanisms underlying the accumulation of G6P, it may serve as a proxy‐measure for accumulation of several osmotically important metabolites and metabolic stress on the exercising muscle.

The absolute concentration of PCr immediately post‐exercise could not be established. However, based on earlier studies using the same type of exercise (Bogdanis et al., 1996; Parolin et al., 1999), it can be assumed that PCr is close to depletion directly after the third interval. As the PCr‐recovery relies on oxidative phosphorylation of ATP, the PCr‐recovery time follows that of citrate‐synthase; the rate‐limiting step for oxidative phosphorylation in the muscle, and the oxidative capacity of the muscle (Bogdanis et al., 1996; McCully et al., 1993). Interestingly, PCr‐recovery was highly correlated with the performance measurements from exercise bouts 2 and 3 but unrelated to perturbation or recovery of pH, Pi or G6P. The strong link between PCr‐recovery and the power output variables as well as the lack of relationship between power output and other muscle metabolites such as pH and Pi has previously been reported from studies using muscle biopsies to assess metabolite content (Bogdanis et al., 1996, 1998). Results showed a strong correlation between power output during the second and third exercise bouts but that was unrelated to the power output during the first bout. It could be argued that power output during bouts 2 and 3 reflects endurance capacity, i.e., the ability to sustain a higher power output over time. This is supported by the identified correlation between power output of the later bouts and PCr‐recovery time post‐exercise. Further support is provided by earlier work showing that resynthesis of PCr after HIIT relates to endurance fitness (Bogdanis et al., 1996). This supports the concept that PCr‐recovery time and power output in exercise bouts 2 and 3 mainly reflects skeletal muscle oxidative capacity. Importantly, no relationship between these variables and plasma volume drop or muscle volume expansion was observed. This indicates that exercise at relatively high workloads for longer duration has less influence on muscle volume expansion or plasma volume drop compared to very high workloads for a shorter duration, which is supported by earlier reports (Convertino et al., 1981). This is corroborated by findings showing that glycogenolysis (and hence accumulation of metabolites) is maximally activated during the first 15 s of the initial exercise bout (Parolin et al., 1999). Also, recent data indicate that additional interval bouts do not result in any additive effects on $\dot{V}O_{2}\text{max}$ (Metcalfe et al., 2012, 2015). Instead, it seems that adding bouts at the expense of the exercise intensity reduces the training effect; this is presumably related to a decrease in maximal work load during each interval (Phillips et al., 2017). The present data provide mechanistic insight into these observations and indicate that metabolic perturbation by decreased glycolytical flux‐rate and accumulation of muscle G6P is a key event in fluid flux between the vascular compartment and muscle tissue. This could also be a plausible stimulus behind the hemodynamic adaption to exercise including the increase in plasma volume and subsequent expansion of the blood volume leading to improvements in $\dot{V}O_{2}\text{max}$.

In the current study, we aimed to estimate the exercise‐induced volume shift into skeletal muscle tissue and to link this fluid shift to exercise‐induced metabolic stress in the tissue. While simultaneous assessments are only possible using MRI, it is necessary to perform the exercise outside the MRI suite. Thus, we acknowledge that the time (90–120 s after completed exercise) to the first measurements includes the re‐synthesis of numerous metabolites such as high energy substrates, i.e., PCr and ATP. It may be suggested that this limits the capacity to characterize the post‐exercise metabolic condition and to relate changes in the metabolic condition within the skeletal muscle with the volume shifts. Nevertheless, the consecutive measurements over time of both volume and metabolites allow for accurate curve‐fitting and estimation of changes over time and the integrated analysis is not dependent on *t*_0_ or fluxes during the actual exercise. Importantly, the estimations that were generated commensurate with what has been reported in invasive studies (Kemp et al., 2007). For future studies, a valuable addition to the literature would be the inclusion of interval protocols of varying intensities and number of sprints in an effort to investigate dose responses.

In summary, this is the first study, to our knowledge, showing that three very brief supramaximal intervals lead to similar acute effects on plasma volume and muscle swelling as more prolonged types of exercise. Our results further identify a relationship between muscle metabolism and muscle volume, with post‐exercise G6P concentration being a good marker for the changes in muscle volume. The current findings support the plausible idea that exercise‐induced reduction of the plasma volume serves as the common primary stimulus underpinning the increase in $\dot{V}O_{2}\text{max}$ seen with both endurance and high‐intensive interval training.

## CONFLICT OF INTEREST

The authors declare no competing interests.

## AUTHOR CONTRIBUTIONS

MM, ER, TG and PS designed the study. MM, PW, TR, MF and ER conducted the experiments and analyzed the data. MM, TG and ER drafted the manuscript. All authors read, revised and approved on text and figures.

## Supporting information



Supplementary Material
