# Supplementary material for: Interval‐induced metabolic perturbation determines tissue fluid shifts into skeletal muscle
Source: Physiol Rep. 2021 Apr 27;9(7):e14841. doi: 10.14814/phy2.14841 (PMC8077120; doi:10.14814/phy2.14841)
Supplement: Supplementary file 1 — Supplementary Material [file PHY2-9-e14841-s001.pdf]

WIMP1

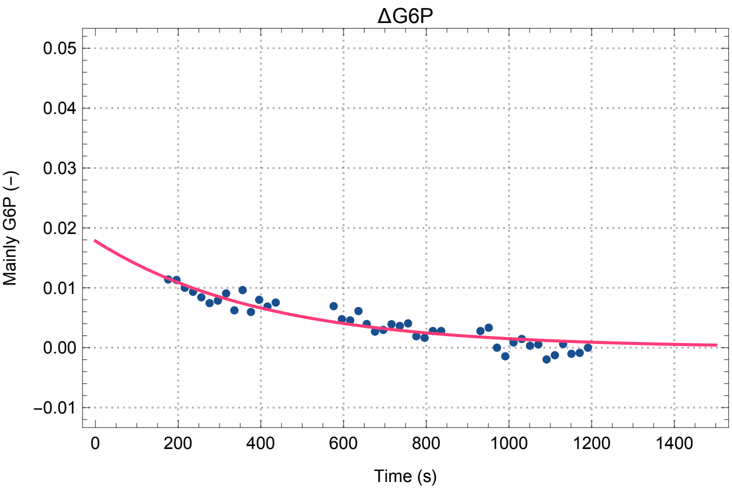

	Estimate	Standard Error	t-Statistic	P-Value
tau	404.684	36.1961	11.1803	7.02685×10^{-14}
g6pHITEnd	0.0178165	0.00150995	11.7994	1.32835×10^{-14}

V_{G6P}: 0.0000440258

1st deltaG6P Post measurement: 0.0113878

WIMP2

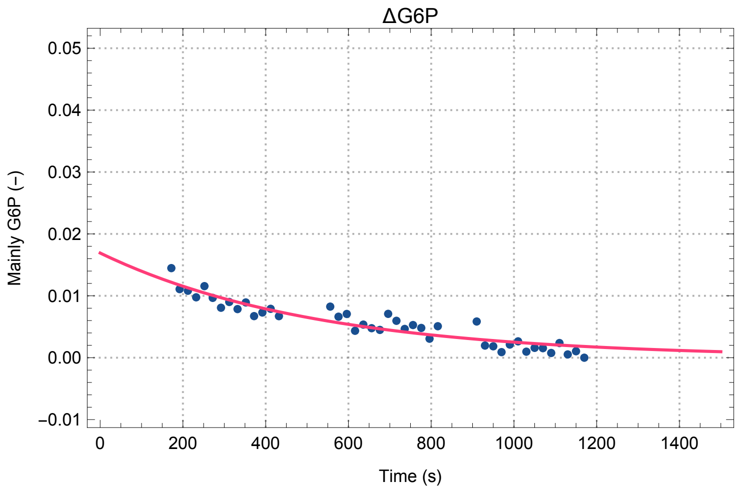

	Estimate	Standard Error	t-Statistic	P-Value
tau	524.523	39.1522	13.397	2.25376×10^{-16}
g6pHITEnd	0.0169114	0.00105502	16.0294	5.19712×10^{-19}

V_{G6P}: 0.0000322414

1st deltaG6P Post measurement: 0.014474

WIMP3

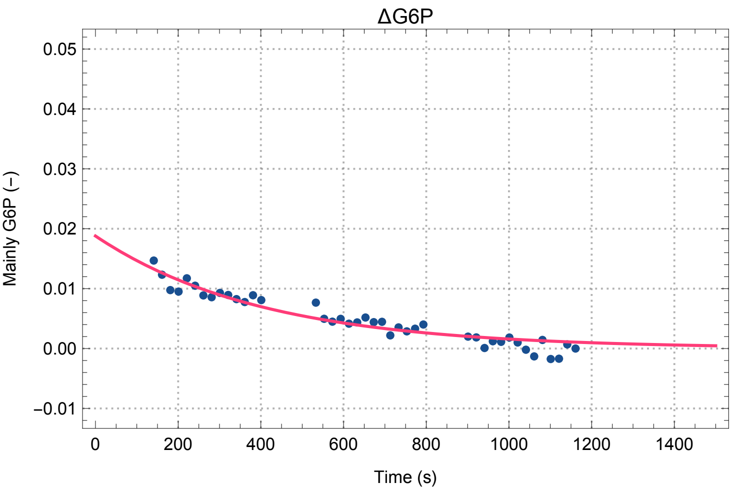

	Estimate	Standard Error	t-Statistic	P-Value
tau	404.653	28.5766	14.1603	3.58142×10^{-17}
g6pHITEnd	0.0187907	0.00115702	16.2406	3.29601×10^{-19}

V_{G6P}: 0.0000464365

1st deltaG6P Post measurement: 0.014688

WIMP4

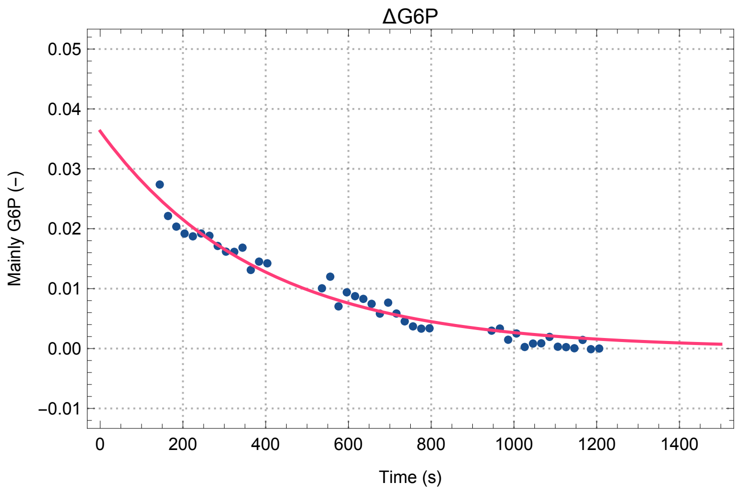

	Estimate	Standard Error	t-Statistic	P-Value
tau	382.701	17.161	22.3006	3.55414×10^{-24}
g6pHITEnd	0.0362888	0.00144989	25.0287	4.75328×10^{-26}

V_{G6P}: 0.0000948228

1st deltaG6P Post measurement: 0.02738

WIMP5

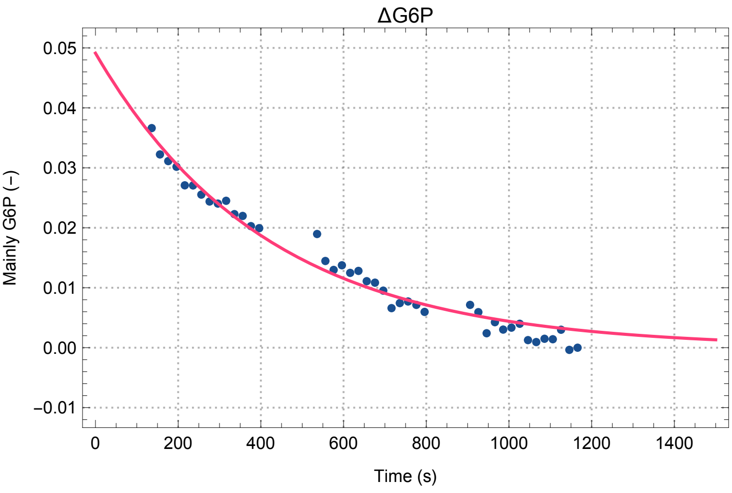

	Estimate	Standard Error	t-Statistic	P-Value
tau	414.477	15.4121	26.893	3.14326×10^{-27}
g6pHITEnd	0.0491265	0.0015555	31.5824	6.7328×10^{-30}

V_{G6P}: 0.000118526

1st deltaG6P Post measurement: 0.036626

WIMP6

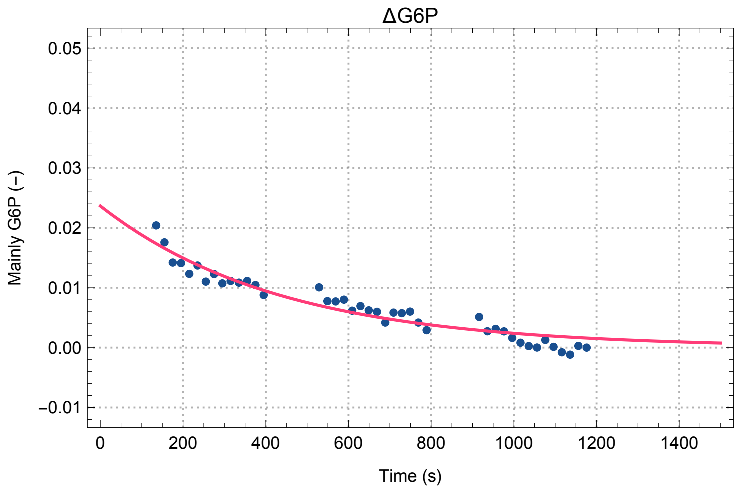

	Estimate	Standard Error	t-Statistic	P-Value
tau	436.275	27.8997	15.6373	1.22524×10^{-18}
g6pHITEnd	0.0236436	0.00125715	18.8073	1.81082×10^{-21}

V_{G6P}: 0.0000541943

1st deltaG6P Post measurement: 0.0204012

WIMP7

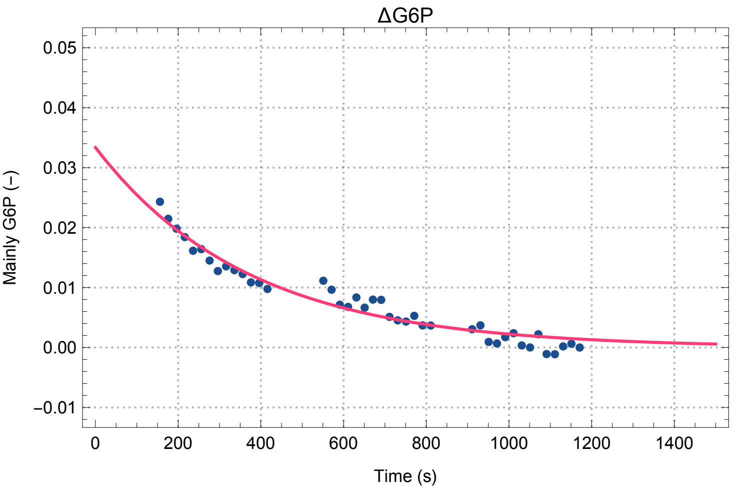

	Estimate	Standard Error	t-Statistic	P-Value
tau	369.996	19.7054	18.7764	1.92137×10^{-21}
g6pHITEnd	0.0333555	0.00166691	20.0104	1.91346×10^{-22}

V_{G6P}: 0.000090151

1st deltaG6P Post measurement: 0.02431

WIMP8

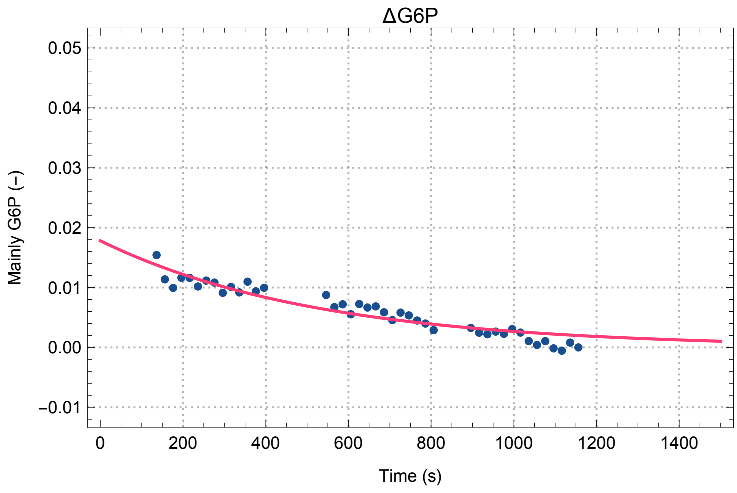

	Estimate	Standard Error	t-Statistic	P-Value
tau	527.221	36.5768	14.4141	1.97168×10^{-17}
g6pHITEnd	0.0178027	0.000958486	18.5737	2.83906×10^{-21}

V_{G6P}: 0.000033767

1st deltaG6P Post measurement: 0.0154194

WIMP9

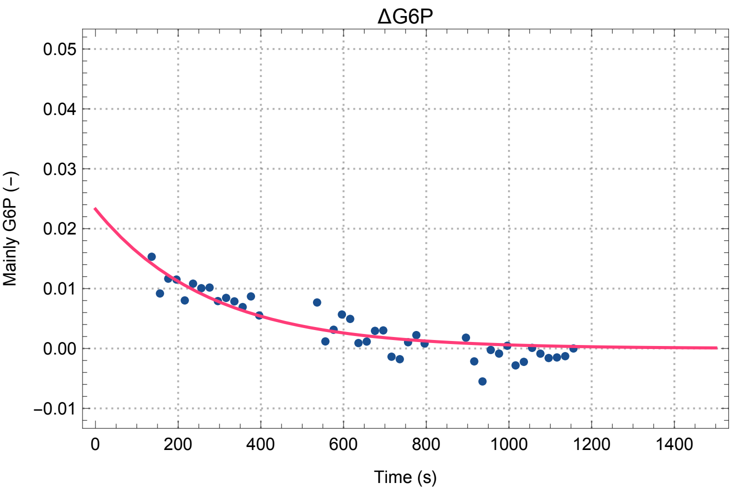

	Estimate	Standard Error	t-Statistic	P-Value
tau	273.172	35.9552	7.59759	2.79803×10^{-9}
g6pHITEnd	0.0232676	0.00306483	7.59183	2.84941×10^{-9}

V_{G6P}: 0.0000851756

1st deltaG6P Post measurement: 0.015315

WIMP10

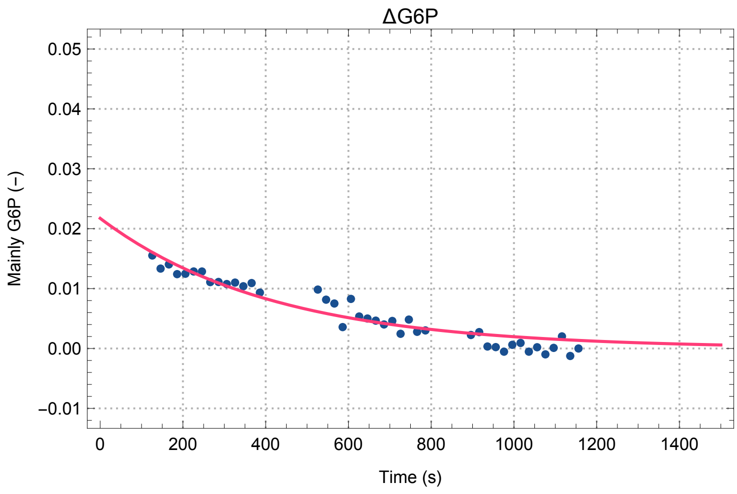

	Estimate	Standard Error	t-Statistic	P-Value
tau	416.17	29.8436	13.945	5.97619×10^{-17}
g6pHITEnd	0.0217301	0.00129307	16.805	9.9667×10^{-20}

V_{G6P}: 0.0000522145

1st deltaG6P Post measurement: 0.0155246

WIMP1

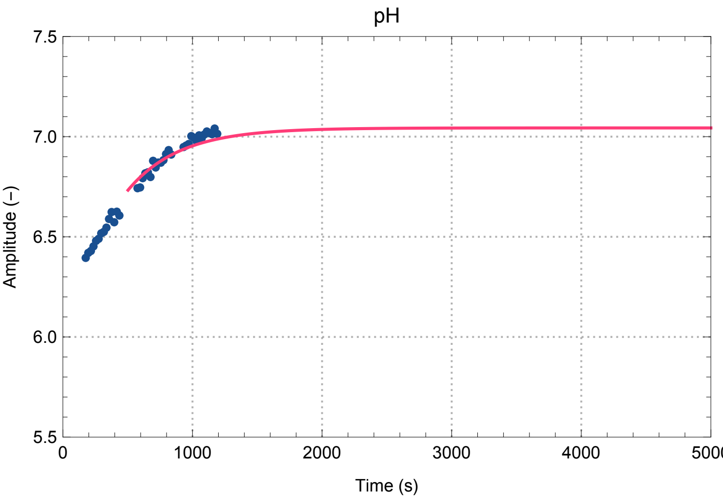

	Estimate	Standard Error	t-Statistic	P-Value
tau	400.626	13.937	28.7454	2.48773×10^{-28}
pHHITEnd	5.9546	0.0360517	165.168	2.56973×10^{-58}

V_{pH}: 0.00271799
Delta pH: 1.0889
pH Pre: 7.0435
1st pH Post estimate: 6.3948

WIMP2

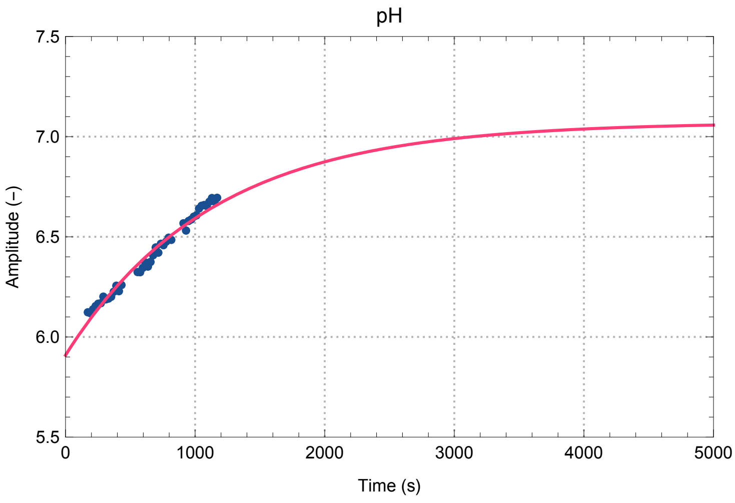

	Estimate	Standard Error	t-Statistic	P-Value
tau	1127.3	29.8106	37.8155	6.29074×10^{-33}
pHHITEnd	5.9086	0.0160101	369.055	2.83957×10^{-72}

V_{pH}: 0.00103166
Delta pH: 1.163
pH Pre: 7.0716
1st pH Post estimate: 6.1231

WIMP3

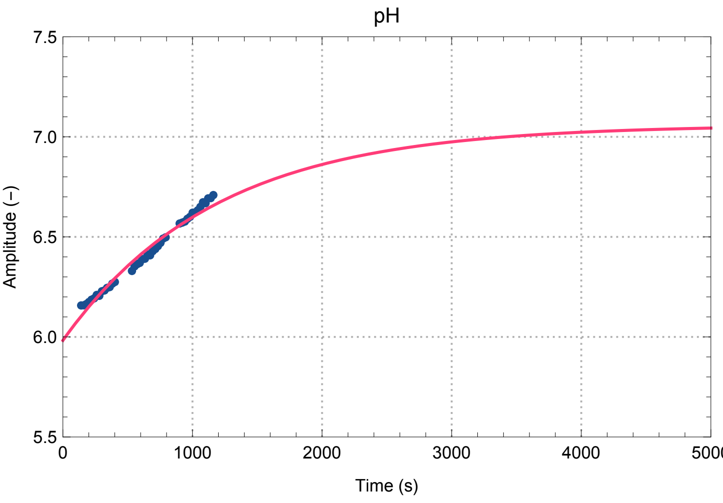

	Estimate	Standard Error	t-Statistic	P-Value
tau	1178.73	31.8305	37.0313	1.42183×10^{-32}
pHHITEnd	5.9826	0.0140925	424.523	1.05068×10^{-74}

V_{pH}: 0.000913357
Delta pH: 1.0766
pH Pre: 7.0592
1st pH Post estimate: 6.1574

WIMP4

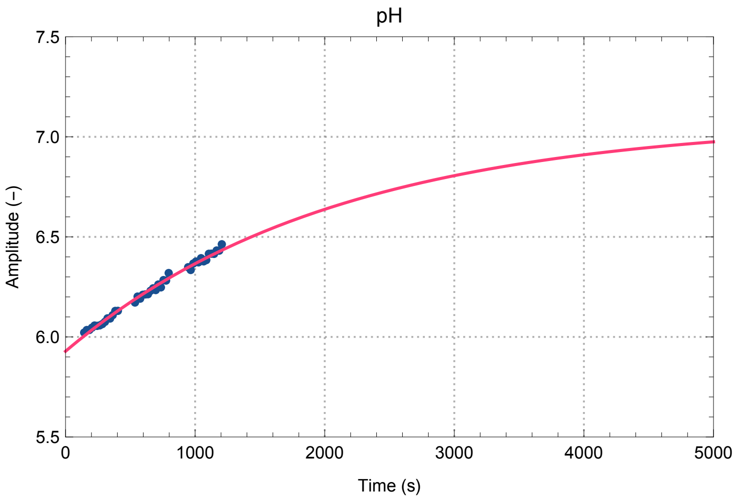

	Estimate	Standard Error	t-Statistic	P-Value
tau	2090.66	30.3096	68.9769	3.31324×10^{-43}
pHHITEnd	5.92821	0.00521335	1137.12	8.07023×10^{-92}

V_{pH}: 0.000551066
Delta pH: 1.15209
pH Pre: 7.0803
1st pH Post estimate: 6.0218

WIMP5

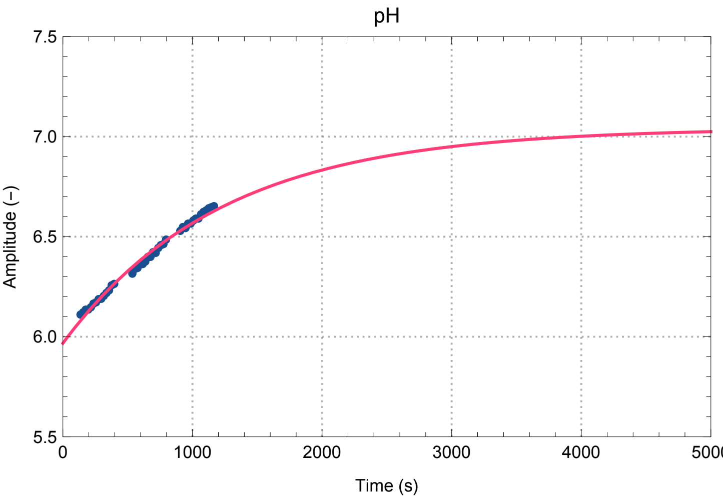

	Estimate	Standard Error	t-Statistic	P-Value
tau	1223.85	20.5329	59.6046	1.07961×10^{-40}
pHHITEnd	5.96752	0.00849504	702.471	1.87743×10^{-83}

V_{pH}: 0.000878761
Delta pH: 1.07548
pH Pre: 7.043
1st pH Post estimate: 6.1108

WIMP6

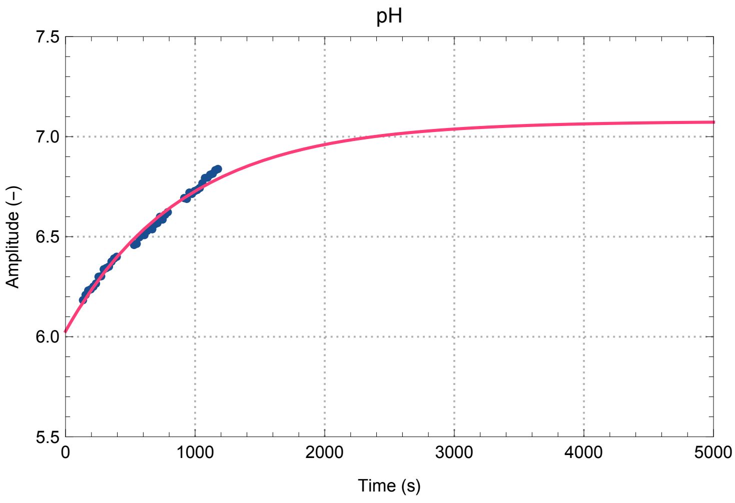

	Estimate	Standard Error	t-Statistic	P-Value
tau	908.19	18.4941	49.107	2.26038×10^{-37}
pHHITEnd	6.02729	0.0123171	489.346	3.57562×10^{-77}

V_{pH}: 0.00115527
Delta pH: 1.04921
pH Pre: 7.0765
1st pH Post estimate: 6.1829

WIMP7

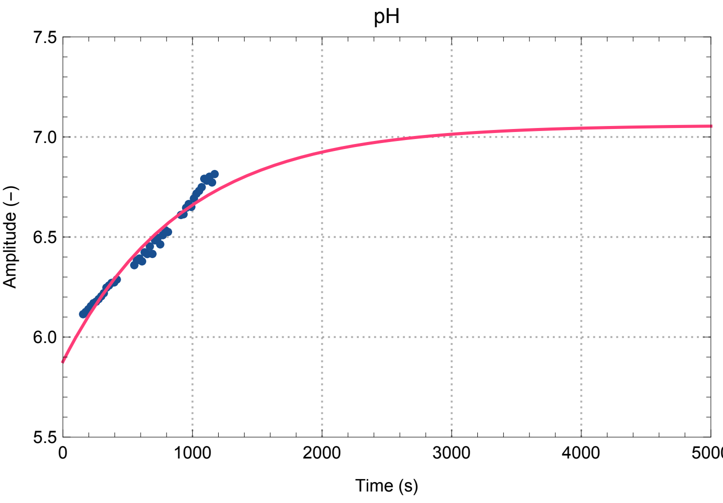

	Estimate	Standard Error	t-Statistic	P-Value
tau	920.884	33.968	27.1103	2.31554×10^{-27}
pHHITEnd	5.87696	0.0257267	228.438	6.05555×10^{-64}

V_{pH}: 0.00128381
Delta pH: 1.18224
pH Pre: 7.0592
1st pH Post estimate: 6.1143

WIMP8

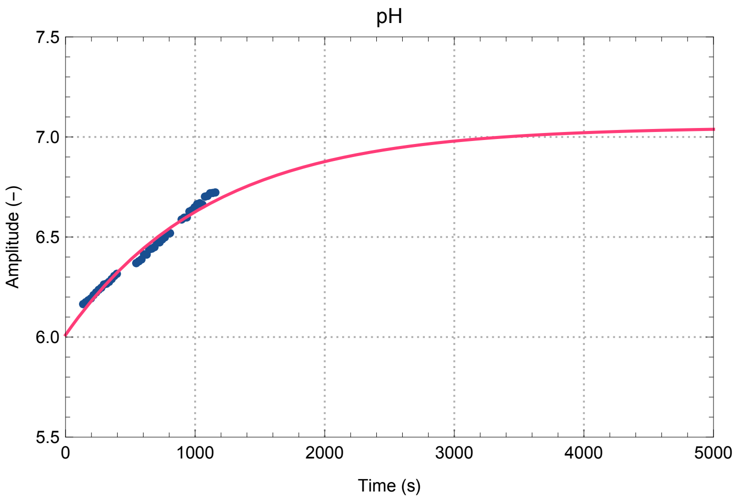

	Estimate	Standard Error	t-Statistic	P-Value
tau	1116.07	30.7999	36.2361	3.30605×10^{-32}
pHHITEnd	6.01048	0.0144858	414.923	2.62247×10^{-74}

V_{pH}: 0.000931503
Delta pH: 1.03962
pH Pre: 7.0501
1st pH Post estimate: 6.1647

WIMP9

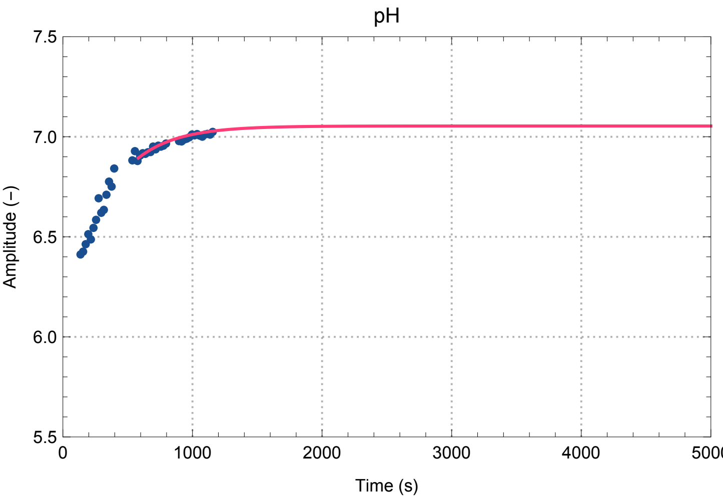

	Estimate	Standard Error	t-Statistic	P-Value
tau	313.855	9.76491	32.1411	3.42653×10^{-30}
pHHITEnd	6.03125	0.0301071	200.327	1.15189×10^{-61}

V_{pH}: 0.00325836
Delta pH: 1.02265
pH Pre: 7.0539
1st pH Post estimate: 6.4119

WIMP10

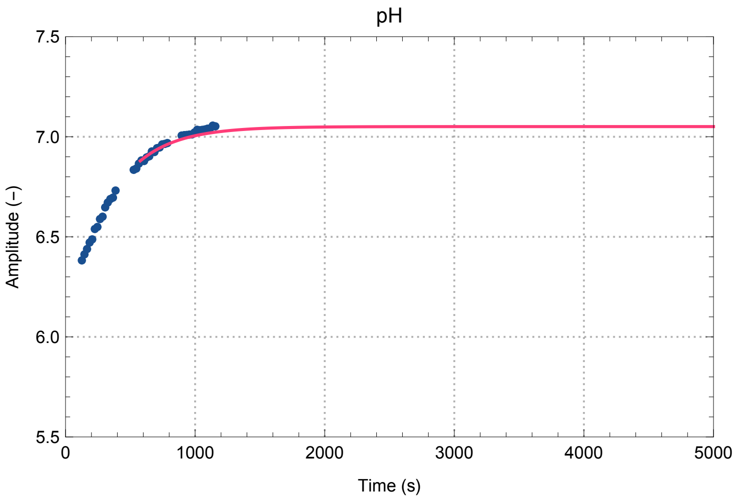

	Estimate	Standard Error	t-Statistic	P-Value
tau	319.559	6.29508	50.7633	6.12061×10^{-38}
pHHITEnd	6.00844	0.0187068	321.19	7.33889×10^{-70}

V_{pH}: 0.00326218
Delta pH: 1.04246
pH Pre: 7.0509
1st pH Post estimate: 6.3823

WIMP1

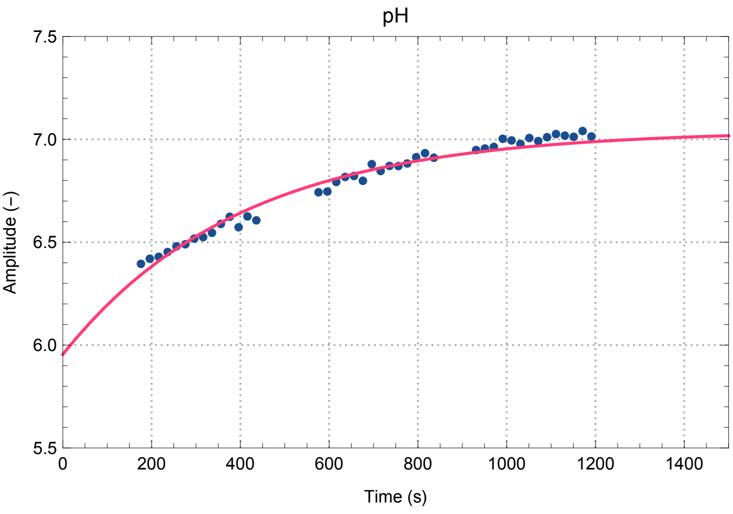

	Estimate	Standard Error	t-Statistic	P-Value
tau	400.626	13.937	28.7454	2.48773×10^{-28}
pHHITEnd	5.9546	0.0360517	165.168	2.56973×10^{-58}

V_{pH}: 0.00271799
Delta pH: 1.0889
pH Pre: 7.0435
1st pH Post estimate: 6.3948

WIMP2

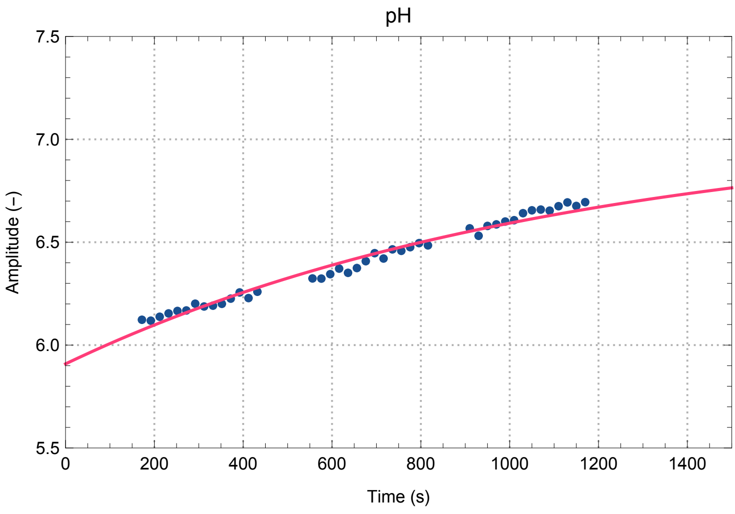

	Estimate	Standard Error	t-Statistic	P-Value
tau	1127.3	29.8106	37.8155	6.29074×10^{-33}
pHHITEnd	5.9086	0.0160101	369.055	2.83957×10^{-72}

V_{pH}: 0.00103166
Delta pH: 1.163
pH Pre: 7.0716
1st pH Post estimate: 6.1231

WIMP3

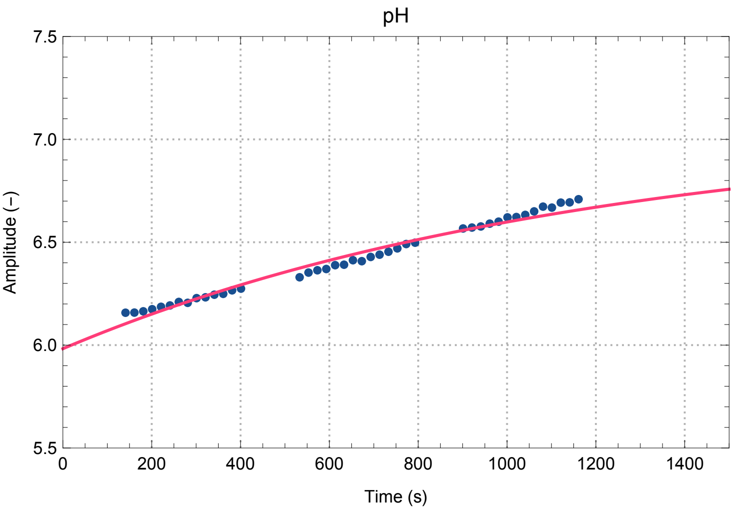

	Estimate	Standard Error	t-Statistic	P-Value
tau	1178.73	31.8305	37.0313	1.42183×10^{-32}
pHHITEnd	5.9826	0.0140925	424.523	1.05068×10^{-74}

V_{pH}: 0.000913357
Delta pH: 1.0766
pH Pre: 7.0592
1st pH Post estimate: 6.1574

WIMP4

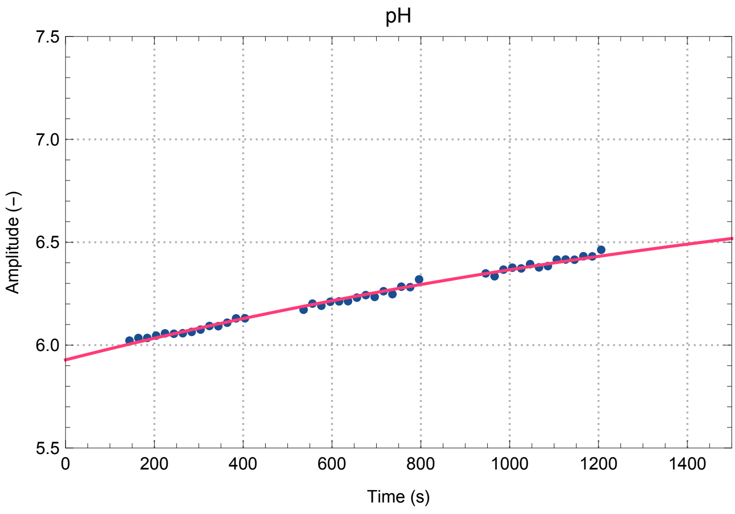

	Estimate	Standard Error	t-Statistic	P-Value
tau	2090.66	30.3096	68.9769	3.31324×10^{-43}
pHHITEnd	5.92821	0.00521335	1137.12	8.07023×10^{-92}

V_{pH}: 0.000551066
Delta pH: 1.15209
pH Pre: 7.0803
1st pH Post estimate: 6.0218

WIMP5

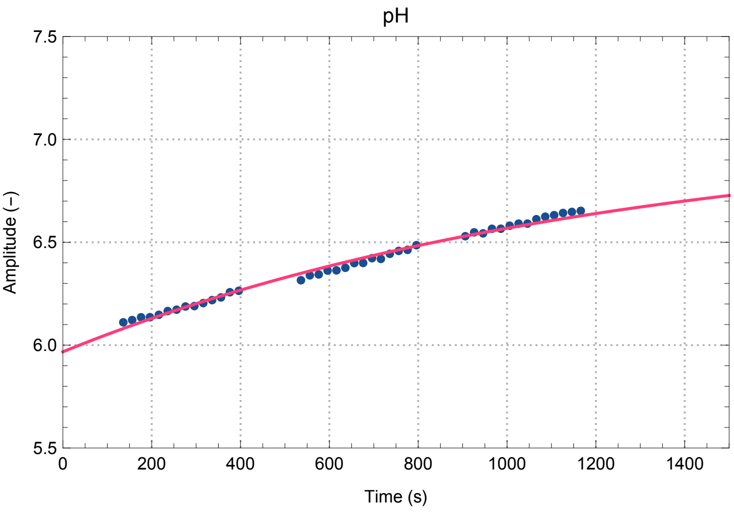

	Estimate	Standard Error	t-Statistic	P-Value
tau	1223.85	20.5329	59.6046	1.07961×10^{-40}
pHHITEnd	5.96752	0.00849504	702.471	1.87743×10^{-83}

V_{pH}: 0.000878761
Delta pH: 1.07548
pH Pre: 7.043
1st pH Post estimate: 6.1108

WIMP6

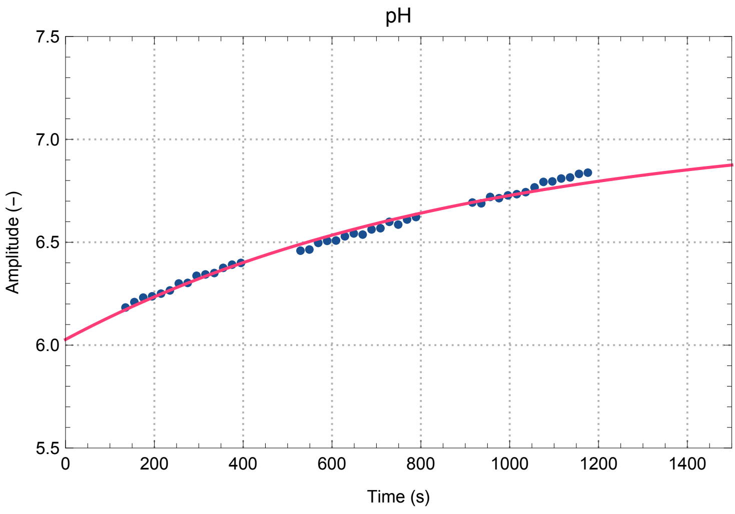

	Estimate	Standard Error	t-Statistic	P-Value
tau	908.19	18.4941	49.107	2.26038×10^{-37}
pHHITEnd	6.02729	0.0123171	489.346	3.57562×10^{-77}

V_{pH}: 0.00115527
Delta pH: 1.04921
pH Pre: 7.0765
1st pH Post estimate: 6.1829

WIMP7

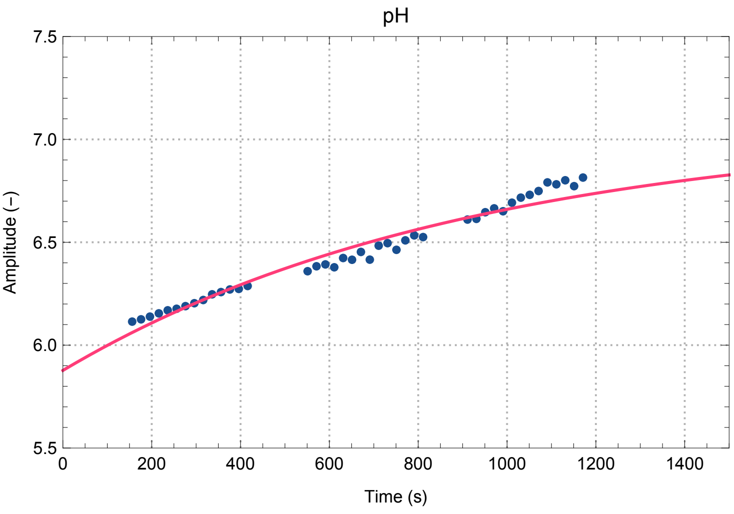

	Estimate	Standard Error	t-Statistic	P-Value
tau	920.884	33.968	27.1103	2.31554×10^{-27}
pHHITEnd	5.87696	0.0257267	228.438	6.05555×10^{-64}

V_{pH}: 0.00128381
Delta pH: 1.18224
pH Pre: 7.0592
1st pH Post estimate: 6.1143

WIMP8

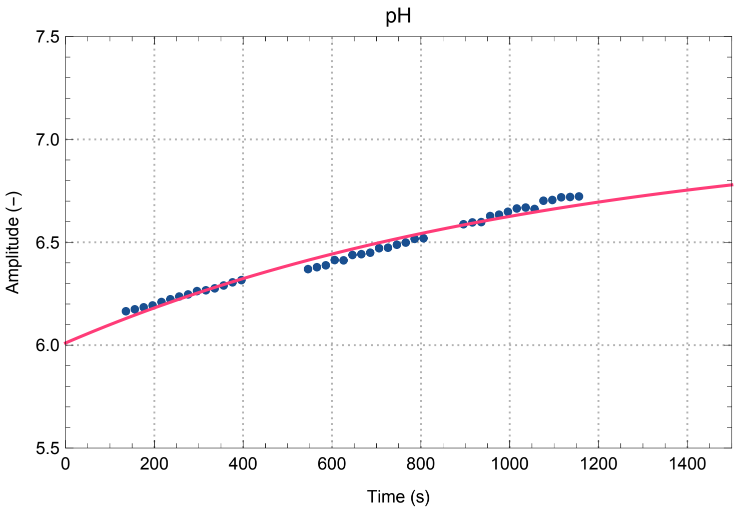

	Estimate	Standard Error	t-Statistic	P-Value
tau	1116.07	30.7999	36.2361	3.30605×10^{-32}
pHHITEnd	6.01048	0.0144858	414.923	2.62247×10^{-74}

V_{pH}: 0.000931503
Delta pH: 1.03962
pH Pre: 7.0501
1st pH Post estimate: 6.1647

WIMP9

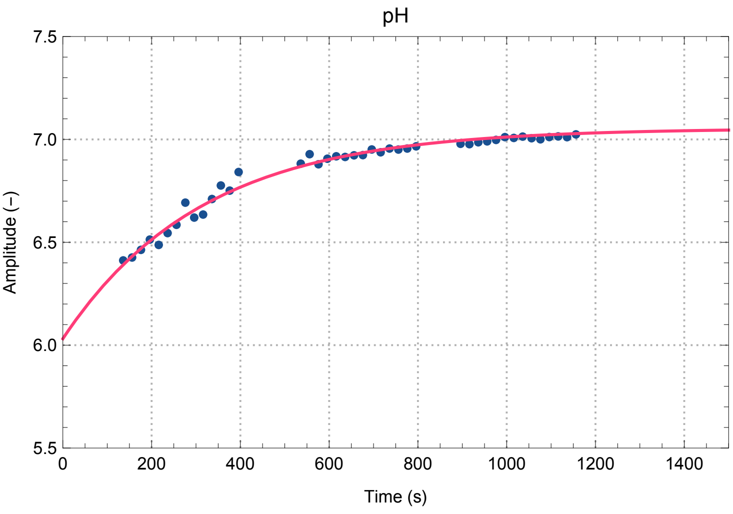

	Estimate	Standard Error	t-Statistic	P-Value
tau	313.855	9.76491	32.1411	3.42653×10^{-30}
pHHITEnd	6.03125	0.0301071	200.327	1.15189×10^{-61}

V_{pH}: 0.00325836
Delta pH: 1.02265
pH Pre: 7.0539
1st pH Post estimate: 6.4119

WIMP10

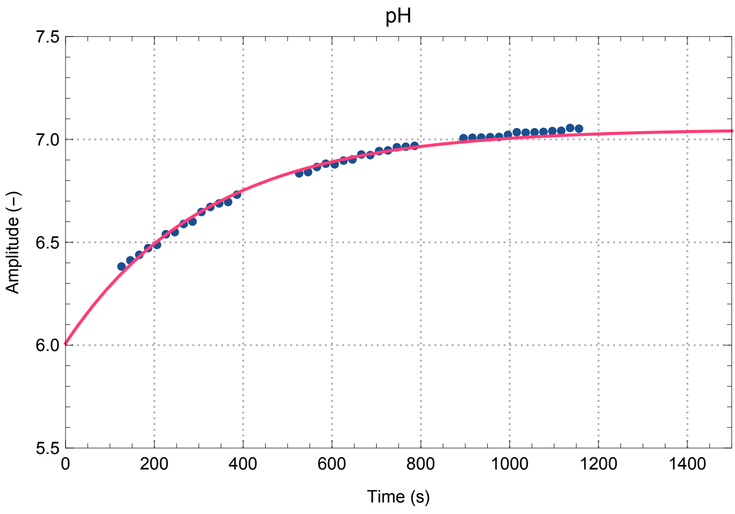

	Estimate	Standard Error	t-Statistic	P-Value
tau	319.559	6.29508	50.7633	6.12061×10^{-38}
pHHITEnd	6.00844	0.0187068	321.19	7.33889×10^{-70}

V_{pH}: 0.00326218
Delta pH: 1.04246
pH Pre: 7.0509
1st pH Post estimate: 6.3823

WIMP1

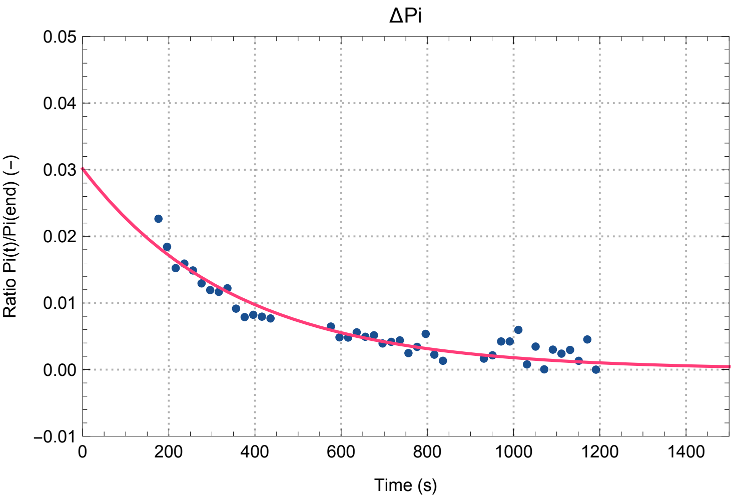

	Estimate	Standard Error	t-Statistic	P-Value
tau	354.07	24.1133	14.6836	1.05433×10^{-17}
piHITEnd	0.0301524	0.00206284	14.6169	1.22998×10^{-17}

V_{Pi}: 0.0000851593

1st deltaPi Post measurement: 0.022643

WIMP2

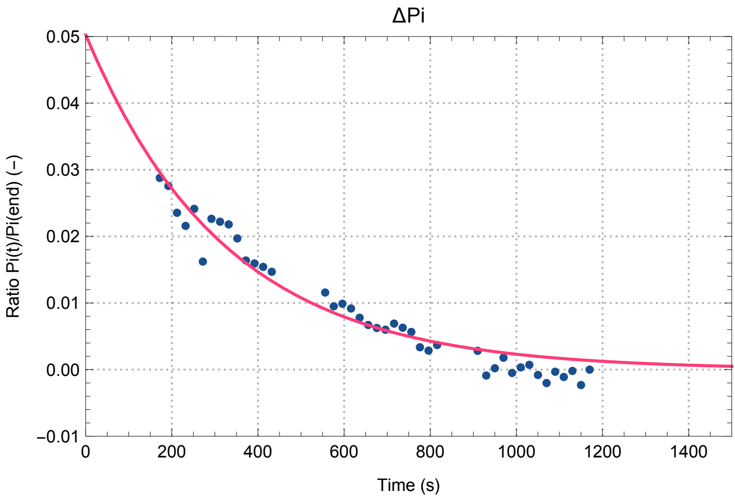

	Estimate	Standard Error	t-Statistic	P-Value
tau	324.571	19.1377	16.9598	7.21787×10^{-20}
piHITEnd	0.0502948	0.00308398	16.3084	2.85003×10^{-19}

V_{Pi}: 0.000154958

1st deltaPi Post measurement: 0.028769

WIMP3

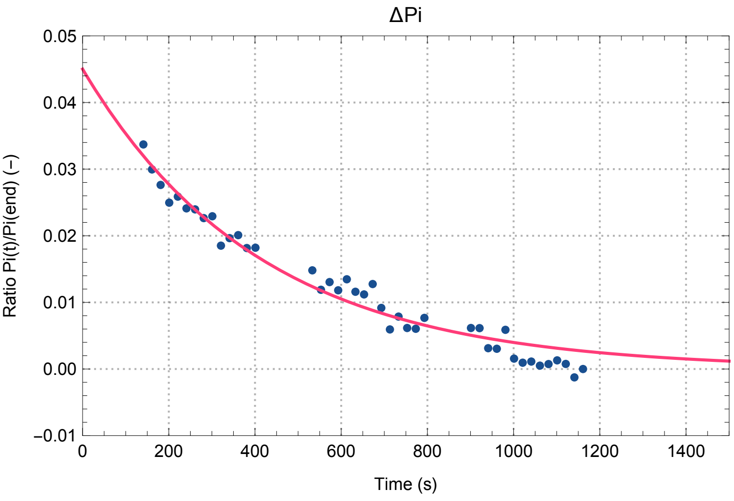

	Estimate	Standard Error	t-Statistic	P-Value
tau	412.402	18.4832	22.3123	3.48567×10^{-24}
piHITEnd	0.0450146	0.0017461	25.7801	1.55758×10^{-26}

V_{Pi}: 0.000109152

1st deltaPi Post measurement: 0.033707

WIMP4

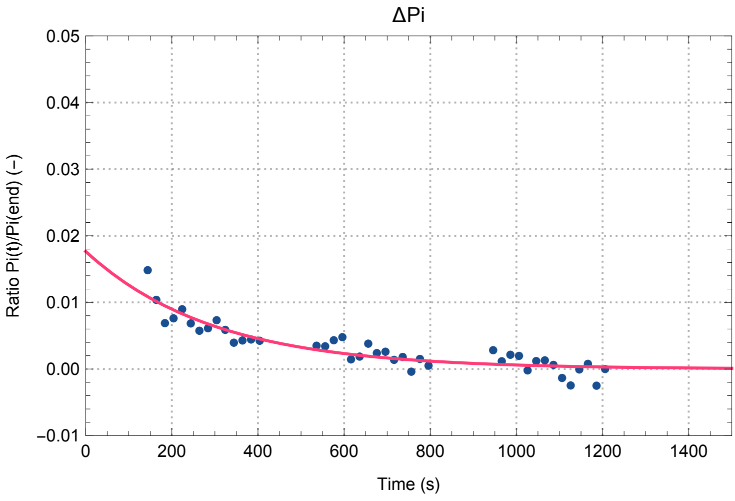

	Estimate	Standard Error	t-Statistic	P-Value
tau	295.846	32.6137	9.07122	2.9711×10^{-11}
piHITEnd	0.0176527	0.00192654	9.1629	2.25763×10^{-11}

V_{Pi}: 0.0000596686

1st deltaPi Post measurement: 0.014831

WIMP5

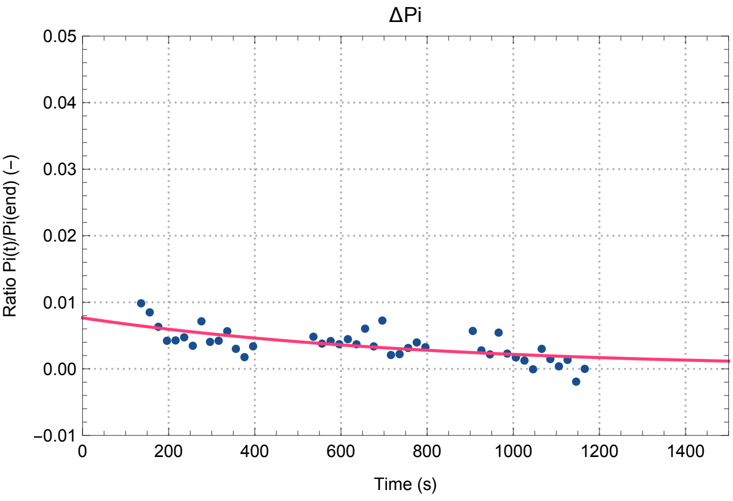

	Estimate	Standard Error	t-Statistic	P-Value
tau	794.32	161.506	4.9182	0.000015361
piHITEnd	0.00765452	0.000977745	7.82875	1.35112×10^{-9}

V_{Pi}: 9.63657×10^{-6}

1st deltaPi Post measurement: 0.009849

WIMP6

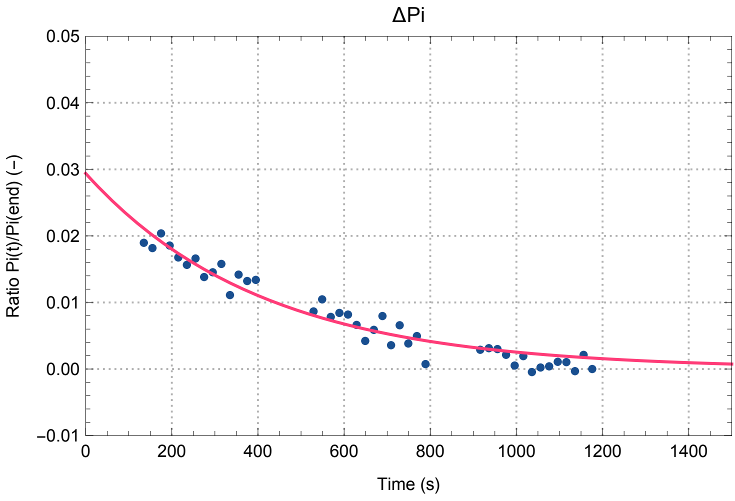

	Estimate	Standard Error	t-Statistic	P-Value
tau	408.329	22.8673	17.8565	1.16256×10^{-20}
piHITEnd	0.0293905	0.00140353	20.9403	3.62922×10^{-23}

V_{Pi}: 0.0000719775

1st deltaPi Post measurement: 0.018964

WIMP7

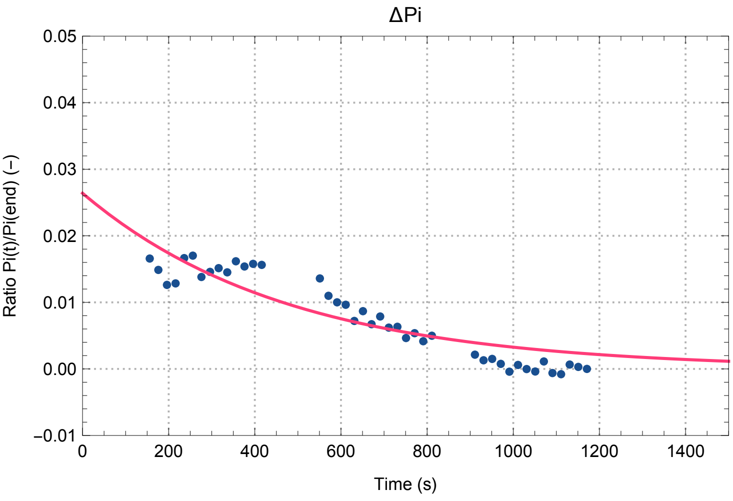

	Estimate	Standard Error	t-Statistic	P-Value
tau	478.412	47.8219	10.004	1.90893×10^{-12}
piHITEnd	0.0263981	0.00222443	11.8673	1.10972×10^{-14}

V_{Pi}: 0.0000551786

1st deltaPi Post measurement: 0.016581

WIMP8

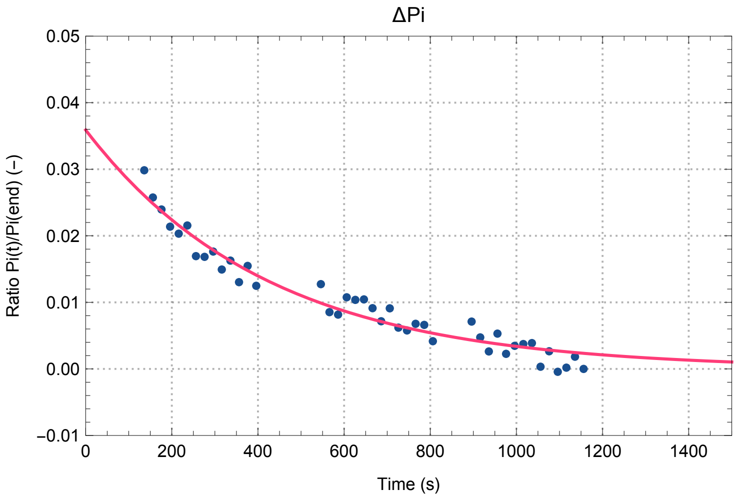

	Estimate	Standard Error	t-Statistic	P-Value
tau	423.565	20.8541	20.3109	1.11053×10^{-22}
piHITEnd	0.035897	0.001495	24.0113	2.25928×10^{-25}

V_{Pi}: 0.0000847496

1st deltaPi Post measurement: 0.029844

WIMP9

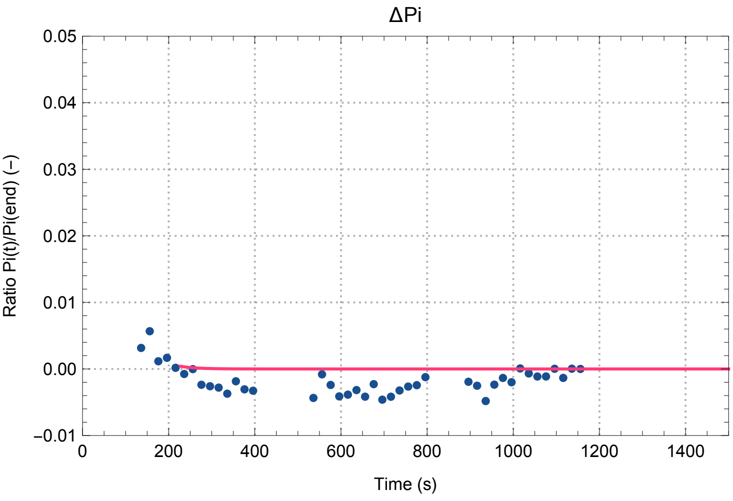

	Estimate	Standard Error	t-Statistic	P-Value
tau	37.5755	36.1847	1.03843	0.305304
piHITEnd	0.172298	0.651851	0.264322	0.792889

V_{Pi}: 0.00458539

1st deltaPi Post measurement: 0.003161

WIMP10

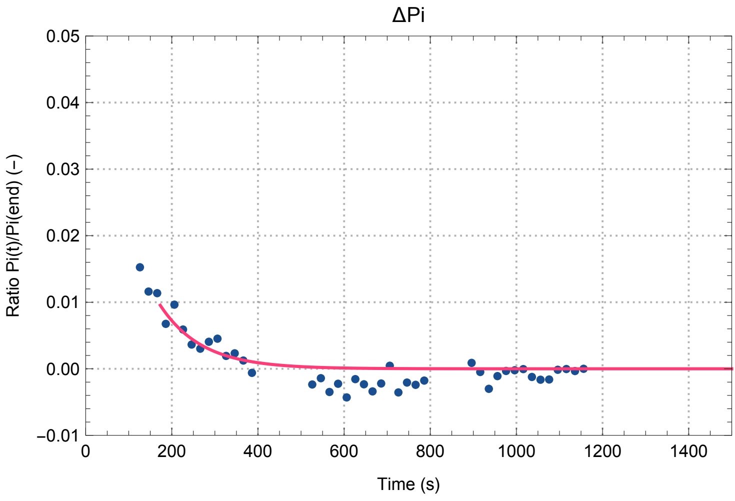

	Estimate	Standard Error	t-Statistic	P-Value
tau	96.854	14.3961	6.72777	4.49757×10^{-8}
piHITEnd	0.0570321	0.0149361	3.8184	0.000457535

V_{Pi}: 0.000588846

1st deltaPi Post measurement: 0.015254
